# Effect of Fusidic Acid and Some Nitrogen-Containing Derivatives on Liposomal and Mitochondrial Membranes

**DOI:** 10.3390/membranes13100835

**Published:** 2023-10-20

**Authors:** Mikhail V. Dubinin, Anna I. Ilzorkina, Elena V. Salimova, Manish S. Landage, Ekaterina I. Khoroshavina, Sergey V. Gudkov, Konstantin N. Belosludtsev, Lyudmila V. Parfenova

**Affiliations:** 1 Department of Biochemistry, Cell Biology and Microbiology, Mari State University, pl. Lenina 1, 424001 Yoshkar-Ola, Russia; 2 Institute of Theoretical and Experimental Biophysics, Russian Academy of Sciences, Institutskaya 3, 142290 Pushchino, Russia; 3 Institute of Petrochemistry and Catalysis, Russian Academy of Sciences, Prospekt Oktyabrya 141, 450075 Ufa, Russia; 4 Prokhorov General Physics Institute of the Russian Academy of Sciences, Vavilov St. 38, 119991 Moscow, Russia

**Keywords:** fusidic acid, liposomes, laurdan, membrane permeability, mitochondria, oxidative phosphorylation, calcium, ROS

## Abstract

The paper assesses the membranotropic action of the natural antibiotic fusidic acid (FA) and its derivatives. It was found that a FA analogue with ethylenediamine moiety (derivative 2), in contrast to native FA and 3,11-dioxime analogue (derivative 1), is able to increase the mobility of the lipid bilayer in the zone of lipid headgroups, as well as to induce permeabilization of lecithin liposome membranes. A similar effect of derivative 2 is also observed in the case of rat liver mitochondrial membranes. We noted a decrease in the microviscosity of the mitochondrial membrane and nonspecific permeabilization of organelle membranes in the presence of this agent, which was accompanied by a decrease in mitochondrial Δψ and OXPHOS efficiency. This led to a reduction in mitochondrial calcium retention capacity. The derivatives also reduced the production of H_2_O_2_ by mitochondria. The paper considers the relationship between the structure of the tested compounds and the observed effects.

## 1. Introduction

Fusidic acid (FA), derived from *Fusidium coccineum*, is a natural 29-nor-protostane triterpene antibiotic. This triterpenoid has a high biological activity against the Gram-positive bacteria *Staphylococcus aureus* and, in particular, strains that are cross-resistant to other antibiotics [1,2]. FA also shows effectiveness against Corynebacteria, Nocardia, anaerobes and Gram-negative Neisseria species [1]. In this regard, it is used in the treatment of the skin, conjunctiva and cornea infections, as well as eye infections caused by atypical microbes [3,4,5]. The action of FA is due to the inhibition of the elongation of the bacterial protein polypeptide chain by selectively binding to the ribosome elongation factor G (EF-G)–GDP complex and preventing its dissociation [6]. It should be noted that eukaryotes also contain several other elongation factors (EF-2), which, however, are not inhibited by FA [7,8].

One of the promising routes for synthetic modifications of natural molecules is the introduction of various nitrogen-containing functional groups, including amines [9] and oximes [10]. It is noted that many biologically active molecules that have amino groups in their structure exhibit membrane-active properties. For example, positively charged diamine moieties are able to electrostatically interact with negatively charged lipids on the surface of bacterial membranes, while the incorporation of bulky hydrophobic substituents into the lipid layer leads to increased membrane permeability, depolarization with disruption of the transmembrane proton gradient, loss of rigidity and cell wall lysis [11,12]. Studies of natural and synthetic compounds containing polyamines have shown that their antimicrobial effect, among other mechanisms of action, may be due to the ability to increase membrane permeability or depolarization [13]. Another class of nitrogen-containing molecules, oximes, have also attracted attention for their anti-inflammatory, antimicrobial, antioxidant, anticancer (and etc.) properties [14]. Synthesis of FA N-substituted analogues was shown to be a promising strategy to obtain active molecules with antimicrobial properties, which not only possess high affinity to EF-G, but also impart additional properties [8,15,16,17]. In particular, it has recently been shown that spermidine and spermine-conjugated FA derivatives are able to be immersed in lipid membranes and exhibit a detergent action. Moreover, spermine-based derivatives formed pores in lipid bilayers, mimicking bacterial membranes. This also contributed to the antibacterial effect of the obtained analogues [8]. Fusidane triterpenoid oximes were shown to effectively inhibit the bacteria growth and proliferation of cancer cells [18]. Based on the fact that oximes have low plasma bioavailability, due to their poor solubility and membrane permeability [9], one would expect that the introduction of nonpolar 29-nor-protostane FA could increase the molecules’ ability to incorporate into the lipid bilayer [19]. All of the above-mentioned prerequisites served as the basis for the selection of FA amine and oxime derivatives as the research objects for the presented study.

Moreover, it is important to note that the membranotropic properties of antibiotics can not only enhance their antibacterial action, but also lead to the appearance of undesirable toxic effects in relation to eukaryotic cells. Triterpenes are well known to accumulate on the membranes of cells and their organelles and, in particular, mitochondria, changing their permeability, as well as significantly modifying the activity of numerous proteins localized primarily in the inner mitochondrial membrane. This, as we have shown previously, is accompanied by a decrease in the efficiency of oxidative ATP synthesis, as well as the hyperproduction of reactive oxygen species (ROS), leading to oxidative stress and various types of cell death [20,21,22,23]. Therefore, the additional verification of modified antibiotics, including FA and its derivatives, is necessary to exclude serious side effects of their use.

**Figure 1:**
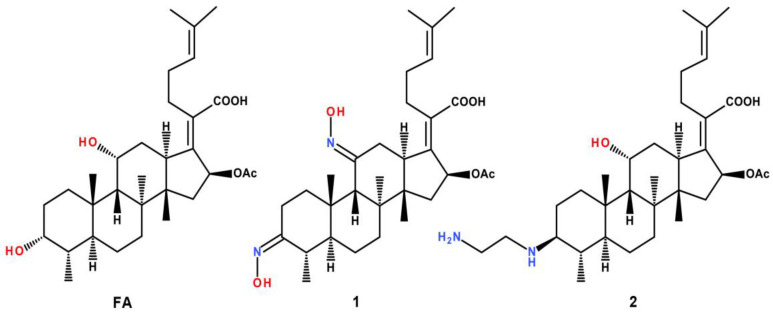
Structure of FA and its derivatives.

In this work, we evaluated the membranotropic properties of FA and its two nitrogen-containing derivatives, dioxime (**1**) and amine (**2**), which we synthesized previously [18,24] (Figure 1), using a model of dipalmitoylphosphatidylcholine (DPPC) and lecithin liposomes that was similar in composition to eukaryotic lipid membranes. Dioxime (compound **1**) showed antibacterial activity towards Gram-positive *S. aureus* (MRSA) with MIC 4.0 μg/mL and cytotoxicity levels similar to those of FA [18], whereas compound **2** showed neither antimicrobial nor antitumor activity [8,25]. Considering the important role of mitochondria in the biological effects of terpenoids, we also evaluated the effects of the tested compounds on major functional parameters of these organelles: (1) the effect of the compounds on the state of the inner mitochondrial membrane and its permeability; (2) membrane potential; (3) respiration and oxidative phosphorylation; (4) the effect of the compounds on the ability of mitochondria to transport and retain Ca^2+^; and (5) ROS production.

## 2. Materials and Methods

### 2.1. Reagents and Chemicals

Inorganic chemicals and medium components were from Sigma-Aldrich (St. Louis, MO, USA). Phosphatidylcholine was from Avanti Polar Lipids (Avanti Polar Lipids Inc., Alabaster, AL, USA). FA (99.3%) was from Hangzhou Hyper Chemicals Limited (Hangzhou, China). Derivatives **1** and **2** were synthesized according to the procedures described in [18,24], correspondingly. FA and its derivatives were prepared as 5 mM stock ethanol solution.

### 2.2. Preparation of Liposomes

Liposomes (large unilamellar vesicles; LUVs) were obtained by a conventional extrusion technique. Ten mg of dry egg phosphatidylcholine (lecithin), or DPPC, was hydrated in 1 mL of buffer for several hours with periodic stirring in a vortex mixer. The buffer contained 10 mM Tris/HCl (pH 8.5), 40 mM KCl and 50 μM EGTA. After five cycles of freezing/thawing, the suspension of multilamellar liposomes was pressed 11 times through a 0.1 μm polycarbonate membrane using an Avanti Micro-Extruder (Avanti Polar Lipids, Birmingham, AL, USA).

### 2.3. Estimation of Laurdan Generalized Polarization (GP)

Laurdan was used for monitoring the phase state of lipid membranes. Its fluorescence (λ_ex_ = 360 nm) was measured using a Varioskan LUX plate reader (Thermo Fisher Scientific, Waltham, MA, USA). Laurdan λ_em_-related blue and red peaks were 440 and 490 nm, respectively. The generalized polarization (GP) (which varies from +1 (most ordered) to −1 (least ordered)) was calculated as GP = (I_440_ − I_490_)/(I_440_ + I_490_), where I_440_ and I_490_ corresponded to emissions at 440 and 490 nm [26,27]. In the experiments with laurdan-containing DPPC liposomes (with a laurdan/lipid molar ratio of 1:200), the suspension of vesicles was added to a buffer containing 40 mM KCl, 50 µM EGTA and 10 mM Tris/HCl (pH 7.5). The final concentration of the lipid was about 50 µM, and laurdan fluorescence was estimated at different temperatures. The typical fluorescence spectra of laurdan in a suspension of DPPC liposomes at 25 °C and 50 °C are shown in Figure S1.

### 2.4. Measurement of Permeabilization of Sulforhodamine B (SRB)-Loaded Liposomes

SRB-loaded LUV were prepared from lecithin by a procedure similar to that described above except that, firstly, the buffer for lipid hydration contained 50 mM SRB instead of 40 mM KCl, and secondly, after extrusion the liposomes were applied to a Sephadex G-50 column to remove the external SRB. The buffer for gel filtration contained 10 mM Tris/HCl (pH 8.5), 50 μM EGTA and 40 mM KCl. The release of SRB was evaluated by the increase in its fluorescence, as described previously [20]. The medium contained 10 mM Tris/HCl (pH 7.5), 50 μM EGTA, and 40 mM KCl. Fluorescence was measured using an Ocean Optics FLAME-T-UV-VIS fiber-optic system (Ocean Optics Inc., Dunedin, FL, USA) (excitation at 565 nm, emission at 586 nm). The total release of the dye was evaluated by the addition of 0.1% Triton X-100. The concentration of SRB in the external buffer was calculated using a calibration curve.

### 2.5. Isolation of Mitochondria from Rat Liver

Mitochondria were isolated from the liver of male Wistar rats using differential centrifugation [28]. All the experimental protocols were approved by the Ethics Committee of Institute of Theoretical and Experimental Biophysics RAS; Approval Code: 4/2023; Approval Date: 8 February 2023. Using sucrose medium (250 mM sucrose, 1 mM EGTA, and 5 mM HEPES/KOH buffer (pH 7.4)), we obtained a suspension of mitochondria containing 60–80 mg protein/mL.

### 2.6. Measurements of Laurdan Generalized Polarization (GP) and Fluidity of the Mitochondrial Membrane

The fluidity of the mitochondrial membrane was estimated by laurdan fluorescence intensity in temperature-controlled (37 °C) 96-well plates using a Varioskan LUX spectrofluorometer. Freshly obtained mitochondria (0.5 mg mitochondrial protein/mL) were incubated in 210 mM mannitol, 70 mM sucrose, 10 μM EGTA, 5 mM succinic acid, 2 μM rotenone and 10 mM HEPES/KOH buffer (pH 7.4), supplemented with 1 μM laurdan. After that, the samples were incubated in the dark for 30 min at 37 °C. Laurdan fluorescence was excited at 360 nm, and the emission was measured at 430 and 490 nm, using a Varioskan LUX plate spectrofluorometer at 37 °C (Thermo Fisher Scientific, Waltham, MA, USA). The generalized polarization (GP) was defined as GP = (I_430_ − I_490_)/(I_430_ + I_490_), where I_430_ and I_490_ are the emission intensities at 430 and 490 nm, respectively [26,27]. The typical fluorescence spectra of laurdan in a mitochondrial suspension at 25 °C and 37 °C are shown in Figure S2. A decrease in the fluorescence intensity of the 430 nm peak with an increase in temperature from 25 °C to 37 °C may indicate a decrease in membrane viscosity and, as a consequence, the possibility to use a probe in a mitochondrial suspension to assess membrane fluidity.

### 2.7. Monitoring of Mitochondrial Swelling

Mitochondrial swelling (0.5 mg mitochondrial protein/mL) was monitored using a Multiskan GO plate reader (Thermo Fisher Scientific, Waltham, MA, USA) at 540 nm. The swelling buffer contained 210 mM mannitol, 70 mM sucrose, 10 μM EGTA and 10 mM HEPES/KOH (pH 7.4) and was supplemented with 2.5 mM glutamate plus 2.5 mM malate or 5 mM succinate (with 1 μM rotenone). For optical microscopy, mitochondrial samples, incubated for 10 min in the absence or presence of derivative **2**, were placed on a glass slide (using 10 μL of mitochondrial suspension) and covered with the cover glass to capture the images using the EVOS M5000 imaging system (Thermo Fisher Scientific).

### 2.8. Monitoring of Mitochondrial Δψ

Tetraphenylphosphonium (TPP^+^) distribution across the inner mitochondrial membrane was used to measure the mitochondrial Δψ. The TPP^+^-concentration was monitored by a Record-4usb electrometrical system, equipped with a TPP^+^-sensitive electrode. Mitochondrial suspension samples (1.0 mg mitochondrial protein/mL) were incubated in 210 mM mannitol, 70 mM sucrose, 10 μM EGTA, 0.5 μM TPP^+^ and 10 mM HEPES/KOH buffer (pH 7.4), supplemented with 2.5 mM glutamate plus 2.5 mM malate or 5 mM succinate (with 1 μM rotenone).

### 2.9. Mitochondrial Respiration and Oxidative Phosphorylation

The O_2_ consumption rate was measured polarographically using an Oxygraph Plus system (Hansatech Instruments, King’s Lynn, UK) [28]. Mitochondrial suspension samples (1.0 mg mitochondrial protein/mL) were incubated in 200 mM sucrose, 20 mM KCl, 5 mM succinate, 1 μM rotenone, 0.5 mM EGTA, 2 mM KH_2_PO_4_ and 10 mM HEPES/KOH buffer (pH 7.4). We monitored respiration in the resting state (or state 2), state 3 (succinate + ADP), state 4 (after ADP exhaustion) and state 3U_DNP_. The respiratory control ratio was estimated as state 3/state 4, according to [29].

### 2.10. Mitochondrial Calcium Transport and Accumulation

Ca^2+^ transport in the mitochondrial suspension samples was estimated colorimetrically using Arsenazo III indicator (675–685 nm) and a Varioskan LUX plate spectrofluorometer [30]. Mitochondrial suspension samples (0.5 mg mitochondrial protein/mL) were incubated in 210 mM mannitol, 70 mM sucrose, 1 mM KH_2_PO_4_, 50 μM Arsenazo III, 10 μM EGTA and 10 mM HEPES/KOH buffer (pH 7.4). The summary of Ca^2+^ pulses, which caused the spontaneous release of an ion from the mitochondrial matrix due to the induction of an mitochondrial permeability transition pore (MPT pore) opening, corresponded to the organelle’s calcium retention capacity (CRC).

### 2.11. H_2_O_2_ Production by Mitochondria

H_2_O_2_ production by mitochondria was monitored using an Amplex Red assay (λ_ex_ = 560 nm; λ_em_ = 590 nm) and a Varioskan LUX plate spectrofluorometer (Thermo Fisher Scientific, Waltham, MA, USA) at 37 °C [31]. Mitochondria samples (0.1 mg mitochondrial protein/mL) were incubated in 210 mM mannitol, 70 mM sucrose, 10 μM EGTA and 10 mM HEPES/KOH buffer (pH 7.4), supplemented with 2.5 mM glutamate plus 2.5 mM malate or 5 mM succinate (with 1 μM rotenone), horseradish peroxidase (1 a.u./mL) and 10 μM Amplex Red.

### 2.12. Statistical Analysis

The data were analyzed using GraphPad Prism 8. The results were presented as means ± SEM. The statistical significance of differences between means was evaluated using a one-way analysis of variance (ANOVA), followed by the Tukey multiple comparison post hoc test. A significance level of *p* < 0.05 was chosen for statistical significance.

## 3. Results

### 3.1. Effect of FA and Its Derivatives on the Phase State and Permeability of Liposomal Membranes

**Figure 2:**
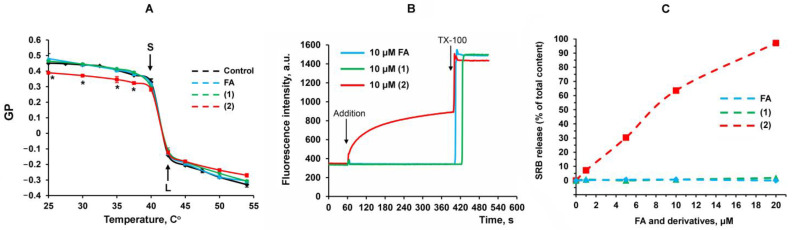
The effect of FA and its derivatives on the state and permeability of the liposomal membrane. (**A**)—Laurdan GP in DPPC liposomes. Liquidus (L) and solidus (S) marks are presented as points of maximum curvature. DPPC concentration is 50 μM. The initial data for the calculation (at 25 °C and 50 °C) are presented in Supplementary Figure S1. * Differences between control (without additions) and experiment (with tested compound **2**) were statistically significant (*p* < 0.05). (**B**)—Effect of 10 μM of FA and compounds **1** and **2** on SRB release from lecithin liposomes at 25 °C. Other additions: 0.1% Triton X-100 (TX-100). Lecithin concentration is 50 μM. (**C**)—effect of increasing test compound concentrations on SRB release from lecithin liposomes at 25 °C. The results are presented as mean ± SEM (*n* = 4).

It is known that FA, and especially its derivatives, are able to integrate into lipid membranes and disrupt the dynamics of phospholipids [4,8]. In this work, we evaluated the effect of FA and its derivatives on the phase behavior and properties of lipid membranes, using a laurdan fluorescent probe. The laurdan generalized polarization (GP) parameter reflects the hydration of lipid bilayer and the mobility of water molecules in the region of the lipid heads [26,27]. Figure 2 shows the temperature dependence of laurdan GP, which makes it possible to estimate the main phase transition in DPPC liposome membranes. Figure 2A shows that 10 μM of FA and its tested derivatives have no effect on the main phase transition in liposomal membranes. On the other hand, it can be seen that derivative **2** reduces the GP parameter of laurdan in liposomes under pre-transition conditions, which may indicate an increase in the mobility of the lipid bilayer in the solid crystalline state.

In this work, we also assessed whether FA and its derivatives are able to influence the membrane permeability of SRB fluorescent probe-loaded lecithin liposomes. Figure 2B,C show that FA and derivative **1** have no effect on the permeability of liposomes for SRB over the entire range of concentrations studied. At the same time, only 1 μM of derivative **2** induces the release of SRB from liposomes, and its half-maximal effect (50% SRB release) is achieved at an agent concentration of about 7.5 μM.

### 3.2. Effect of FA and Its Derivatives on the Permeability and Phase State of Mitochondrial Membranes

**Figure 3:**
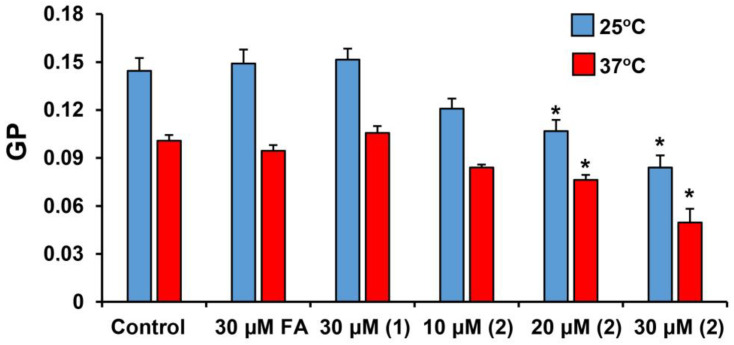
Effect of FA and its derivatives on laurdan GP in mitochondria at 25 and 37 °C. The results are presented as means ± SEM (*n* = 4). * *p* < 0.05 (versus control, 30 μM FA and 30 μM derivative **1**). The initial data for the calculation are presented in Supplementary Figure S2.

Mitochondrial membranes are one of the targets of triterpenoids in cells. Previously, we revealed the ability of triterpenoids and their derivatives to induce non-specific permeabilization, not only of the artificial membrane of liposomes, but also of the membranes of these organelles, which was also accompanied by the effect of the compounds on the overall fluidity of the membrane lipid bilayer [20,23,32]. Using laurdan, we also evaluated the effect of compounds on the state of mitochondrial membranes (Figure 3). One can see that FA and derivative **1**, at a maximum concentration of 30 μM, have no effect on the GP parameter in the mitochondrial membranes. On the other hand, derivative **2** dose-dependently reduces the GP parameter of laurdan. This means a decrease in the microviscosity of the mitochondrial membrane; it is also associated with an increase in the mobility of water molecules in the zone of lipid heads in the presence of derivative **2**.

**Figure 4:**
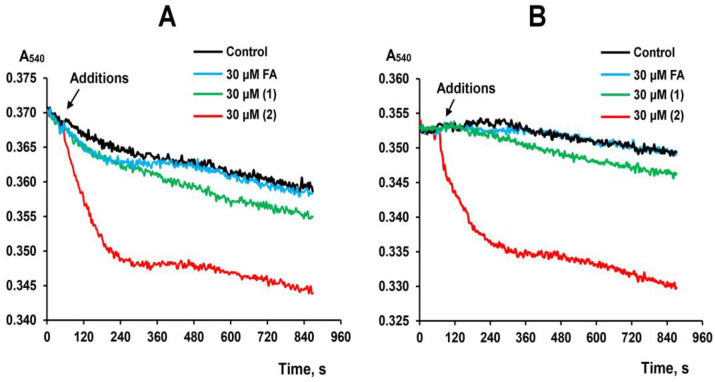
Effect of FA and its derivatives on the permeability of mitochondrial membranes. Typical curves showing the swelling of liver mitochondria induced by tested compounds in the presence of glutamate + malate (**A**) or succinate + rotenone (**B**) at 25 °C. Similar curves were obtained in three independent experiments.

In addition, we evaluated the ability of FA and its derivatives to induce mitochondrial membrane permeability. It is known that the induction of nonspecific permeability of the inner membrane of mitochondria, incubated in a sucrose medium, leads to swelling of the organelles, and this is associated with a decrease in the optical density of the mitochondrial suspension [33,34]. Figure 4A,B show that FA and derivative **1**, at the maximum tested concentration of 30 μM, had practically no effect on the optical density of the suspension of rat liver mitochondria. On the other hand, derivative **2** causes a decrease in the optical density of the mitochondrial suspension. This effect is observed when organelles are energized by the substrates of both complex I and complex II of the respiratory chain and may indicate swelling of the organelles due to the induction of nonspecific permeability of their inner membranes. It is known that a decrease in the optical density of a mitochondrial suspension can also be due to the aggregation of organelles, as has been shown in the case of other triterpenoids [20]. However, in our case, we did not detect mitochondrial aggregation in the presence of derivative **2** (Supplementary Figure S3).

### 3.3. Effect of FA and Its Derivatives on the Δψ of Rat Liver Mitochondria and Parameters of Oxidative Phosphorylation

**Figure 5:**
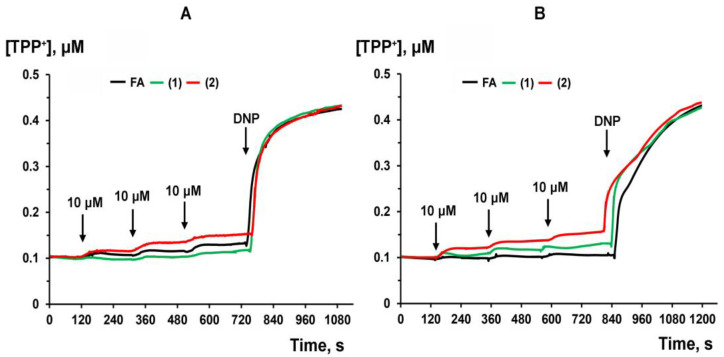
Effect of FA and its derivatives on the membrane Δψ of glutamate/malate—(**A**)-, or succinate—(**B**)-fueled rat liver mitochondria. Maximum reduction in Δψ was achieved with 50 μM DNP. The measurements were carried out at 25 °C. Typical curves recorded with the same sample during the same experiment. Similar curves were obtained in two independent experiments.

In the next part of the work, we evaluated the effect of FA and its derivatives on the functioning of rat liver mitochondria. One can see that FA and derivative **1** do not significantly affect the membrane potential of rat liver mitochondria fueled by glutamate/malate (substrates of ETC complex I) or succinate (a substrate of ETC complex II) + rotenone (Figure 5). However, only 30 µM of derivative **2** significantly reduced Δψ by 17.3 ± 0.8% and 17.8 ± 1.0% when mitochondria were energized with glutamate/malate or succinate, respectively (*n* = 3, *p* < 0.05). We evaluated the effect of derivative **2** on the parameters of succinate-driven respiration of organelles. Table 1 shows that the FA analogue with ethylenediamine moiety does not affect the rate of ADP-stimulated respiration in liver mitochondria or the maximum respiration rate achieved by the addition of protonophore uncoupler 2,4-dinitrophenol (DNP). At the same time, we noted a dose-dependent increase in the rate of respiration of organelles in state 2 and state 4. This action of derivative **2** is also accompanied by a decrease in the respiratory control ratio, reflecting the degree of coupling of respiration and phosphorylation in mitochondria.

### 3.4. The Effect of FA and Its Derivatives on the Transport of Calcium Ions and Hydrogen Peroxide Production by Mitochondria

**Figure 6:**
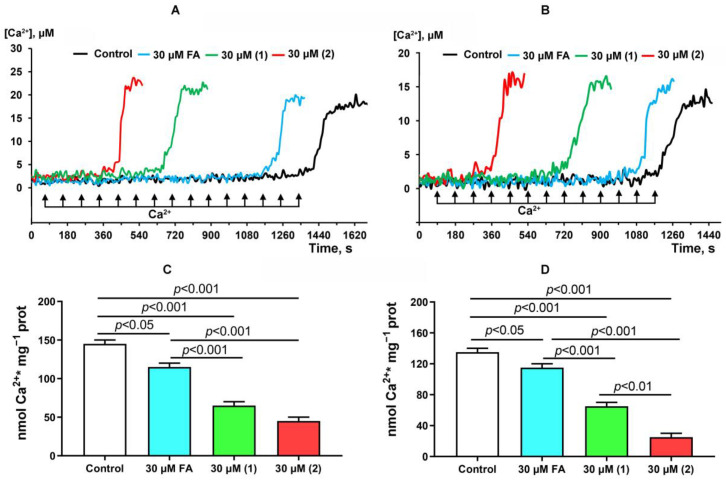
Effect of FA and its derivatives on calcium transport and MPT pore induction in rat liver mitochondria. (**A**,**B**)—Ca^2+^ retention by glutamate/malate- or succinate-fueled rat liver mitochondria in the presence and absence of FA and its derivatives. The figures show the spontaneous release of calcium after successive additions of Ca^2+^ doses (per 5 µM). The measurements were carried out at 25 °C. (**C**,**D**)—Ca^2+^ retention capacity of rat liver mitochondria fueled by glutamate/malate (**C**) or succinate (**D**) in the presence and absence of FA and its derivatives. The results are presented as means ± SEM (*n* = 4).

In the final part of the work, we evaluated the effect of FA and its derivatives on one of the main functions of mitochondria, associated with the transmembrane transport of calcium ions and ion accumulation in the matrices of organelles. Figure 6 shows that rat liver mitochondria are capable of absorbing pulsed additions of calcium ions (per 5 μM), while critical Ca^2+^ overload of the matrix is accompanied by spontaneous release of the ion, indicating the induction of the calcium-dependent MPT pore. Based on the amount of absorbed Ca^2+^ supplements, it is possible to determine the parameter of mitochondrial calcium retention capacity, which characterizes the resistance of organelles to the induction of nonspecific permeability of organelle membranes. FA and its derivatives **1** and **2** cause a decrease in the amount of Ca^2+^ additions leading to the opening of the MPT pore, which is also characterized by a significant decrease in the calcium retention capacity parameter. At the same time, FA derivatives are more effective than the parental compound. Thus, it can be assumed that FA and, especially, its tested derivatives are able to reduce the ability of mitochondria to absorb and retain calcium ions in the matrix.

**Figure 7:**
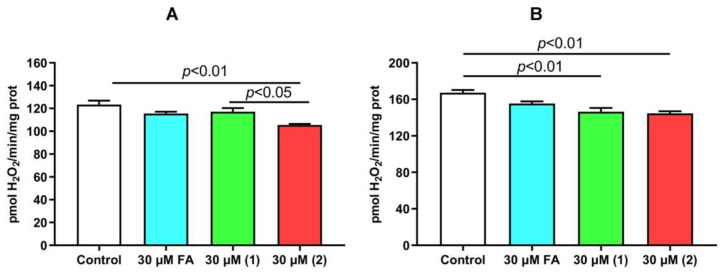
The rate of H_2_O_2_ generation by glutamate/malate—(**A**)- or succinate—(**B**)-fueled rat liver mitochondria in the presence of FA and its derivatives. The measurements were carried out at 37 °C. The results are presented as means ± SEM (*n* = 4).

Mitochondria are also one of the main ROS producers in the cell [35]. Triterpenes have a significant effect on this function of organelles, which affects the redox status of the cell. Therefore, we evaluated the effect of FA and its derivatives on the hydrogen peroxide production by rat liver mitochondria. Figure 7 shows that FA, at a maximum concentration of 30 μM, does not affect the production of H_2_O_2_ by mitochondria, when the organelles are energized by both the substrate of complex I of the respiratory chain and substrate of complex II. On the other hand, derivatives **1** and **2** at the indicated concentration cause a significant decrease in H_2_O_2_ generation. In this case, derivative **2** is slightly more effective under conditions of mitochondrial energization with glutamate/malate.

## 4. Discussion

The search for and development of promising antimicrobial drugs and methods for their use is an urgent problem of modern chemistry and medicine. FA is one of the well-known natural antibiotics used for the treatment of staphylococcal infections (both local and systemic forms) [36]. It is believed that FA is able to be efficiently distributed in various tissues, has low toxicity, and is also characterized by a lack of cross-resistance with other antimicrobials [37]. Currently, attempts are being made to expand the spectrum of antibiotic activity of FA through chemical modification. In a recent work, it was shown that a number of polyamine derivatives of FA, on the one hand, retain the ability to inhibit protein synthesis in bacterial cells by blocking the elongation factor EF-G and, on the other hand, acquire membranotropic properties, demonstrating detergent effects, as well as the ability to form channels in lipid bilayers, mimicking bacterial membranes [8]. This may enhance the antibacterial effect of new FA derivatives. Additionally, these modifications in the structure of FA contributed to an increase in its toxic effect in relation to eukaryotic cells [8].

The results of this work suggest that the toxic effect of new FA derivatives may be due to the effect on the phase state and membrane permeability of eukaryotic cells. Indeed, it can be seen that a FA analogue with ethylenediamine moiety (derivative **2**), in contrast to native FA and derivative **1**, is a powerful inducer of nonspecific membrane permeability of lecithin liposomes, whose lipid composition is close to the membranes of eukaryotic cells. At the same time, experiments on synthetic DPPC liposomes containing laurdan suggest that this effect of derivative **2** is due to an increase in the mobility of the lipid bilayer in the zone of lipid heads, but is not associated with a change in the main phase transition.

Similar effects of derivative **2** are also observed in the case of mitochondrial membranes, which are the target of many membrane-active compounds, including various classes of triterpenoids. In this case, we also noted the induction of nonspecific membrane permeability of isolated rat liver mitochondria in the presence of derivative **2**, which suggests a decrease in the microviscosity of organelle membranes, as evidenced by a decrease in laurdan GP. This effect of derivative **2**, apparently, also causes a decrease in the membrane potential of organelles. It can be seen that this effect is accompanied by an increase in the rate of respiration of organelles in states 2 and 4, which may be due to the uncoupling effect of derivative **2**, based on the permeabilization of the inner mitochondrial membrane. This effect causes the inhibition of OXPHOS in mitochondria, which is also evidenced by a decrease in the respiratory control ratio. It should be noted that, according to known data, FA is only able to suppress the efficiency of oxidative phosphorylation in mitochondria at concentrations of about 0.5 mM [38].

We have also shown that all tested compounds affect the ability of liver mitochondria to transport and accumulate Ca^2+^ in the matrix of organelles, which play an important role in regulating the homeostasis of this ion in eukaryotic cells [39]. FA and, in particular, derivatives **1** and **2,** reduced the CRC of mitochondria, contributing to the induction of the MPT pore phenomenon in the inner membrane of organelles. In addition, derivatives **1** and **2** were able to reduce the generation of hydrogen peroxide by mitochondria, indicating their antioxidant properties. Thus, FA and its derivatives are able to affect ion channels and mitochondrial proteins with different efficiency, leading to changes in the function of organelles.

One could assume that the differences in the effects of FA and its tested derivatives may be related to the chemical structure of the compounds. The transition in the series FA–compound **1**–compound **2** is accompanied by the replacement of -OH groups in the native molecule by oxime =N-OH and diaminoethane -NH(CH_2_)_2_NH_2_ groups, which primarily affects their polarity, hydrophobicity, charge and, accordingly, their behavior in lipid membranes. The theoretical estimation of the lipophilicity of compounds logP (clogP), made by MarvinSketch 20.12.0 [40], showed that the hydrophobicity of the compounds decreases in the following order: derivative **1**, FA and derivative **2** (the logP for each are 5.52, 4.26 and 3.83, respectively), which is in accordance with the values of the dipole moments of the corresponding functional groups [41]. It should be noted that all the studied compounds are quite hydrophobic, so it can be assumed that all of them will be preferentially distributed into membranes. At the same time, FA and derivative **1**, being the least polarized and most hydrophobic, are able to be more effectively distributed into the depth of the lipid bilayer. It was previously shown that the hydrophobicity of FA also determines its effectiveness as an antibacterial agent, since its specific target, EF-G, is predominantly located in the region of the lipid bilayer, increasing the likelihood of their interaction [19]. At the same time, the hydrophobicity of derivative **1** and its interaction with the lipid bilayer of the membrane, apparently, also determines its more pronounced antibacterial effect, as shown in a recent study [18]. Moreover, studies of the biological effects of membrane-active compounds have shown that molecules that disrupt the integrity of the membrane are usually lipophilic and positively charged [11]. Theoretical assessment of pKa and the isoelectric point, carried out using the MarvinSketch 20.12.0 program [40], showed that under the experimental conditions (pH 7.4–7.5), the carboxyl group of the FA is almost completely deprotonated (pKa (-COOH) = 4.7, pI = 2.2), which corresponds to the charge of the molecule being -1 (see Supplementary Figure S4). The corresponding values for compound **1** (pKa(-COOH) = 4.6, pI = 3.5) also indicates the existence of this molecule in the deprotonated form at a neutral pH, i.e., the charge of the molecule is −1 (see Supplementary Figure S5). For ethanediamine derivative **2**, the theoretical values were as follows: pKa(-COOH) = 4.3, pKa(-NH_2_) = 7.3, pKa(-NH-) = 10.5, pI = 8.9. Therefore, in a neutral medium, the molecule exists in both cationic form (-NH_3_^+^, -NH_2_^+^-, -COO^−^) and neutral state (-COO^−^, -NH_2_^+^-). The total charge of the molecule at a pH of 7.4 is estimated to be positive, at a level of 0.42 (see Supplementary Figure S6). As a result, the positive charge of molecule **2** apparently determines its membrane activity.

It can be assumed that the more hydrophilic derivative **2** interacts with the membrane only in the zone of lipid heads. This can be evidenced by the data with laurdan, obtained both on liposomes and mitochondria. Such an effect can significantly reduce its antibacterial activity compared to FA [8], but can lead to the induction of membrane permeability, in particular mitochondrial membranes. This may be facilitated by the positive charge of derivative **2**, leading to its accumulation in the inner mitochondrial membrane, due to the presence of a potential gradient. Moreover, we cannot exclude the accumulation of derivative **2** in the mitochondrial matrix, driven by Δψ (negative inside). It is interesting to note that a similar picture was observed in the case of positively charged ceramide derivatives, which led to nonspecific permeabilization of the inner mitochondrial membrane, the swelling of organelles and the dissipation of Δψ [42]. A similar effect of derivative **2** also contributes to a decrease in the membrane potential of organelles, the inhibition of OXPHOS, and, possibly, a mild uncoupling effect, leading to a decrease in the level of ROS. On the other hand, long-term permeabilization of mitochondrial membranes, as well as the impaired function of calcium ion transport in these organelles in the presence of the tested compounds (especially derivative **2**), may have a toxic effect on eukaryotic cells, which was shown in recent work [8].

## 5. Conclusions

The results obtained show that the biological activity and effectiveness of FA, and its amine and oxime derivatives, may be partially due to the membranotropic effects of the compounds. Depending on their structure, hydrophobicity and charge, they are able to change the surface properties of lipid membranes and the activity of membrane proteins, and to cause permeabilization of the lipid bilayer. Thus, further targeted modification of these compounds may be associated with fine regulation of hydrophobicity and charge, which, on the one hand, will ensure correct binding to the main FA target (bacterial EF-G) but, on the other hand, will not contribute to significant permeabilization of eukaryotic cell membranes and the toxic effect on the host organism. This will allow the creation of new effective and safe antibacterial agents, based on the well-known FA structure.

## Figures and Tables

**Table 1:** Effect of derivative **2** on the succinate-driven O_2_ consumption of rat liver mitochondria.

Additions	State 2	State 3	State 4	State 3U_DNP_	RC
	nmol O_2_ ∗ min^−1^ ∗ mg^−1^ Protein				rel. un.
Control	15.95 ± 0.47	42.52 ± 0.61	18.22 ± 0.60	46.41 ± 0.76	2.34 ± 0.05
20 μM derivative 2	16.50 ± 0.67	42.95 ± 0.56	21.66 ± 0.33 *	48.53 ± 0.26	1.99 ± 0.05 *
30 μM derivative 2	18.09 ± 0.20 *	42.47 ± 0.99	22.55 ± 0.31 *	47.31 ± 0.33	1.92 ± 0.05 *

The measurements were carried out at 25 °C. Table shows means ± SEM (*n* = 3). * *p* < 0.05 (versus control).

## Data Availability

The data presented in this study are available upon request from the corresponding author.

## Supplementary Materials

The following supporting information can be downloaded at: https://www.mdpi.com/article/10.3390/membranes13100835/s1; Figure S1: laurdan emission spectra (λ_ex_ = 360 nm) in DPPC liposomes in the absence (control) and presence of tested compounds at 25 °C (A) and 50 °C (B). Figure S2: Emission spectra of rat liver mitochondria (λ_ex_ = 360 nm) in the absence and presence of laurdan and tested compounds at 25 °C (A) and 37 °C (B). Figure S3: Light microscopy images of rat liver mitochondria in the absence (A) and presence of 30 μM derivative 2 (B). Typical images are presented in two sets for each experimental condition. Images obtained at 25 °C. Scale bar is 20 μm. Figure S4: Theoretical values of pKa and pI for FA. Figure S5: Theoretical values of pKa and pI for compound **1**. Figure S6: Theoretical values of pKa and pI for compound **2**.
