# Supplementary material for: Effect of Fusidic Acid and Some Nitrogen-Containing Derivatives on Liposomal and Mitochondrial Membranes
Source: Membranes (Basel). 2023 Oct 20;13(10):835. doi: 10.3390/membranes13100835 (PMC10608686; doi:10.3390/membranes13100835)
Supplement: Supplementary file 1 [file membranes-13-00835-s001.zip › membranes-2593667-supplementary.pdf]

membranes

Supplementary Material: Effect of Fusidic Acid and Some Nitrogen-Containing Derivatives on Liposomal and Mitochondrial Membranes

Mikhail V. Dubinin, Anna I. Ilzorkina, Elena V. Salimova, Manish S. Landage, Ekaterina I. Khoroshavina, Sergey V. Gudkov, Konstantin N. Belosludtsev, and Lyudmila V. Parfenova

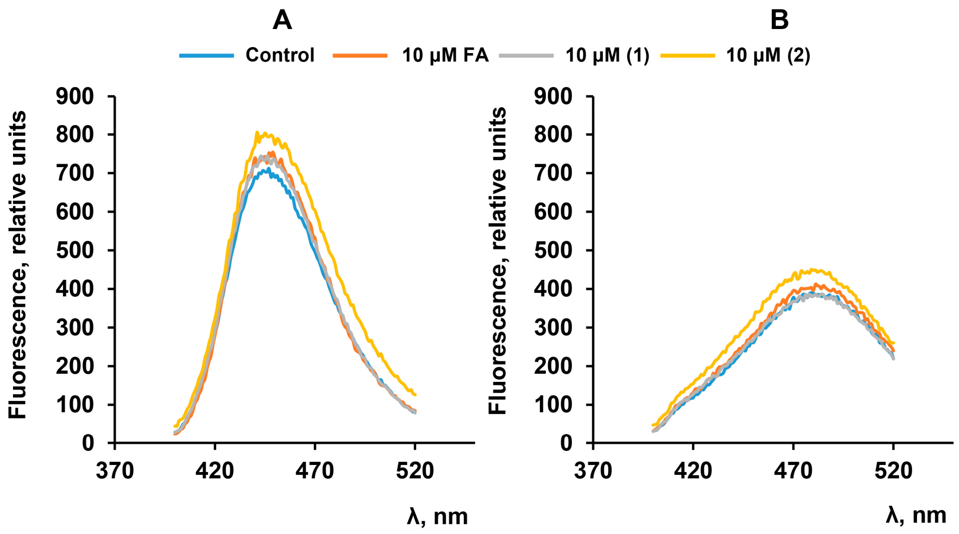

Figure S1. Laurdan emission spectra (λ_{ex} = 360 nm) in DPPC liposomes in the absence (control) and presence of tested compounds at 25 °C (A) and 50°C (B).

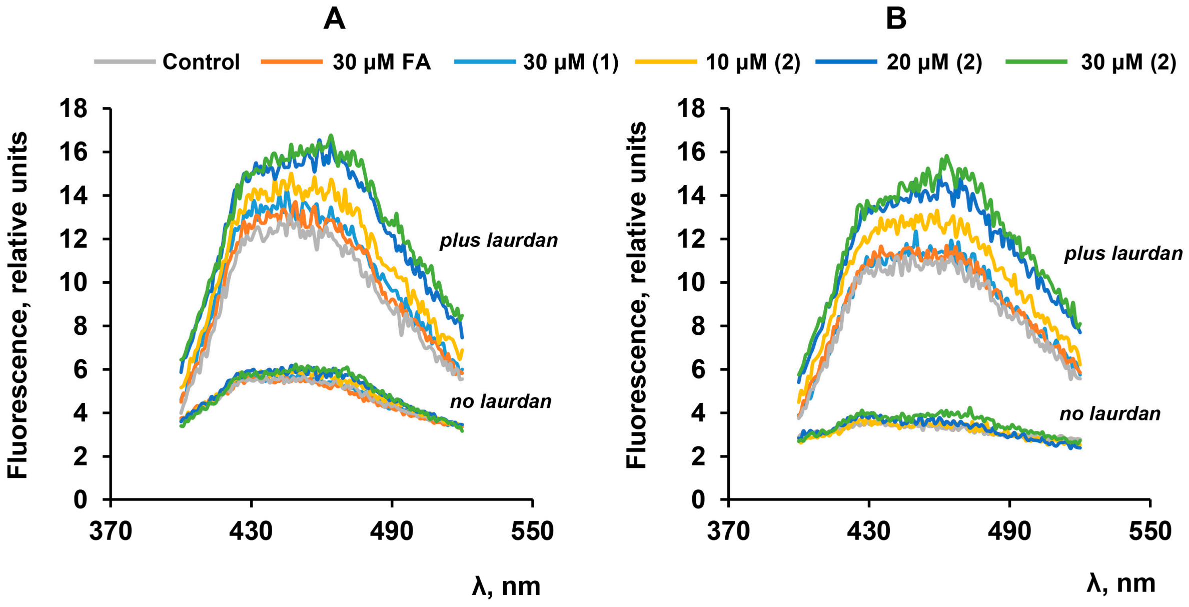

Figure S2. Emission spectra of rat liver mitochondria (λ_{ex} = 360 nm) in the absence and presence of laurdan and tested compounds at 25 °C (A) and 37°C (B).

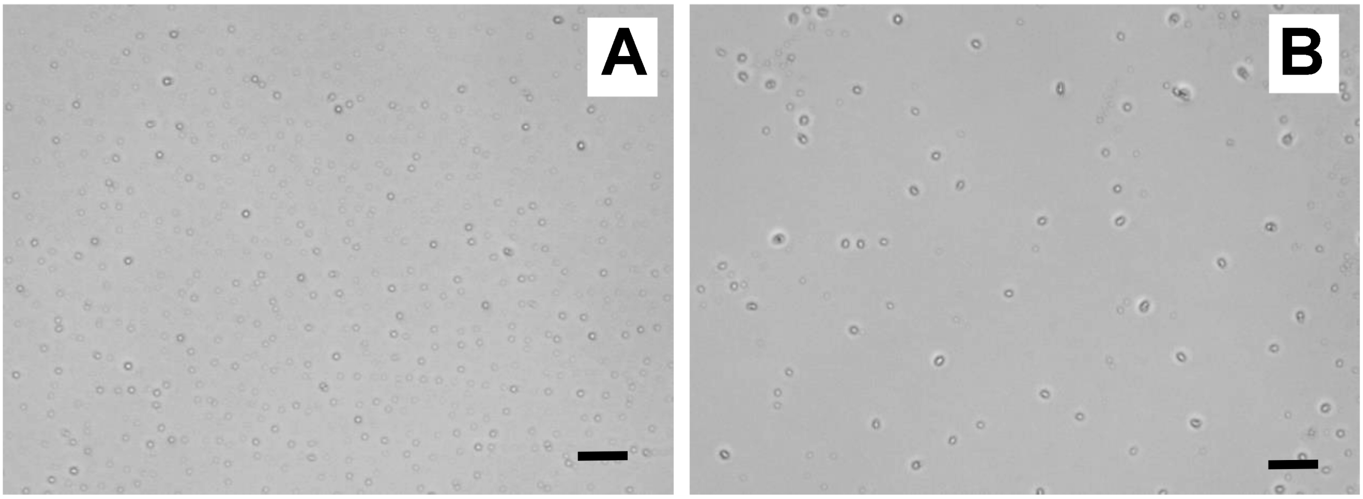

Figure S3. Light microscopy images of rat liver mitochondria in the absence (A) and presence of 30 μM derivative 2 (B). Typical images are presented in two sets for each experimental condition. Images obtained at 25 °C. Scale bar is 20 μm.

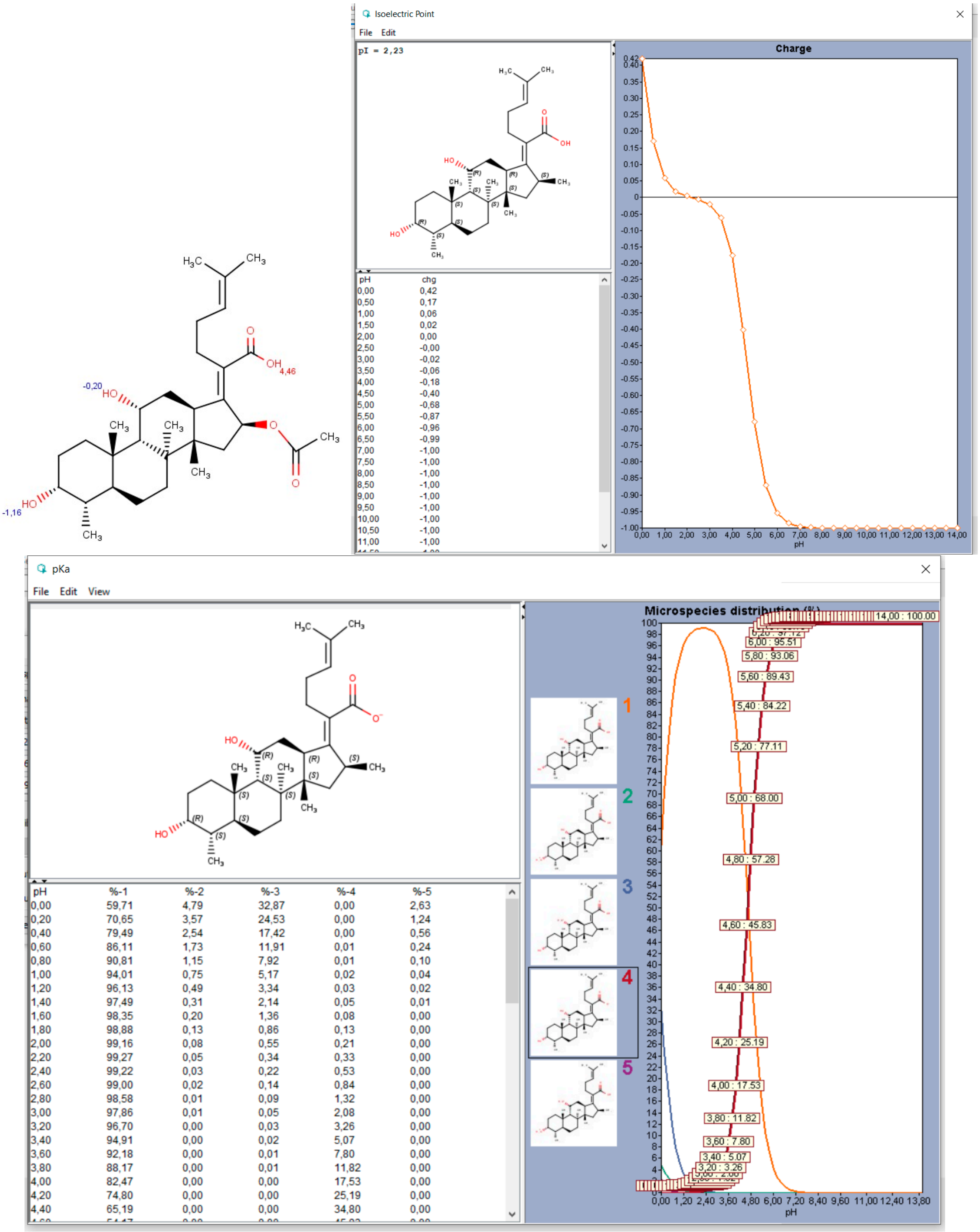

pH	chg
0,00	0,42
0,50	0,17
1,00	0,06
1,50	0,02
2,00	0,00
2,50	-0,00
3,00	-0,02
3,50	-0,06
4,00	-0,18
4,50	-0,40
5,00	-0,68
5,50	-0,87
6,00	-0,96
6,50	-0,99
7,00	-1,00
7,50	-1,00
8,00	-1,00
8,50	-1,00
9,00	-1,00
9,50	-1,00
10,00	-1,00
10,50	-1,00
11,00	-1,00

pH	%-1	%-2	%-3	%-4	%-5
0,00	59,71	4,79	32,87	0,00	2,63
0,20	70,65	3,57	24,53	0,00	1,24
0,40	79,49	2,54	17,42	0,00	0,56
0,60	86,11	1,73	11,91	0,01	0,24
0,80	90,81	1,15	7,92	0,01	0,10
1,00	94,01	0,75	5,17	0,02	0,04
1,20	96,13	0,49	3,34	0,03	0,02
1,40	97,49	0,31	2,14	0,05	0,01
1,60	98,35	0,20	1,36	0,08	0,00
1,80	98,88	0,13	0,86	0,13	0,00
2,00	99,16	0,08	0,55	0,21	0,00
2,20	99,27	0,05	0,34	0,33	0,00
2,40	99,22	0,03	0,22	0,53	0,00
2,60	99,00	0,02	0,14	0,84	0,00
2,80	98,58	0,01	0,09	1,32	0,00
3,00	97,86	0,01	0,05	2,08	0,00
3,20	96,70	0,00	0,03	3,26	0,00
3,40	94,91	0,00	0,02	5,07	0,00
3,60	92,18	0,00	0,01	7,80	0,00
3,80	88,17	0,00	0,01	11,82	0,00
4,00	82,47	0,00	0,00	17,53	0,00
4,20	74,80	0,00	0,00	25,19	0,00
4,40	65,19	0,00	0,00	34,80	0,00

Figure S4. Theoretical values of pKa and pI for FA.

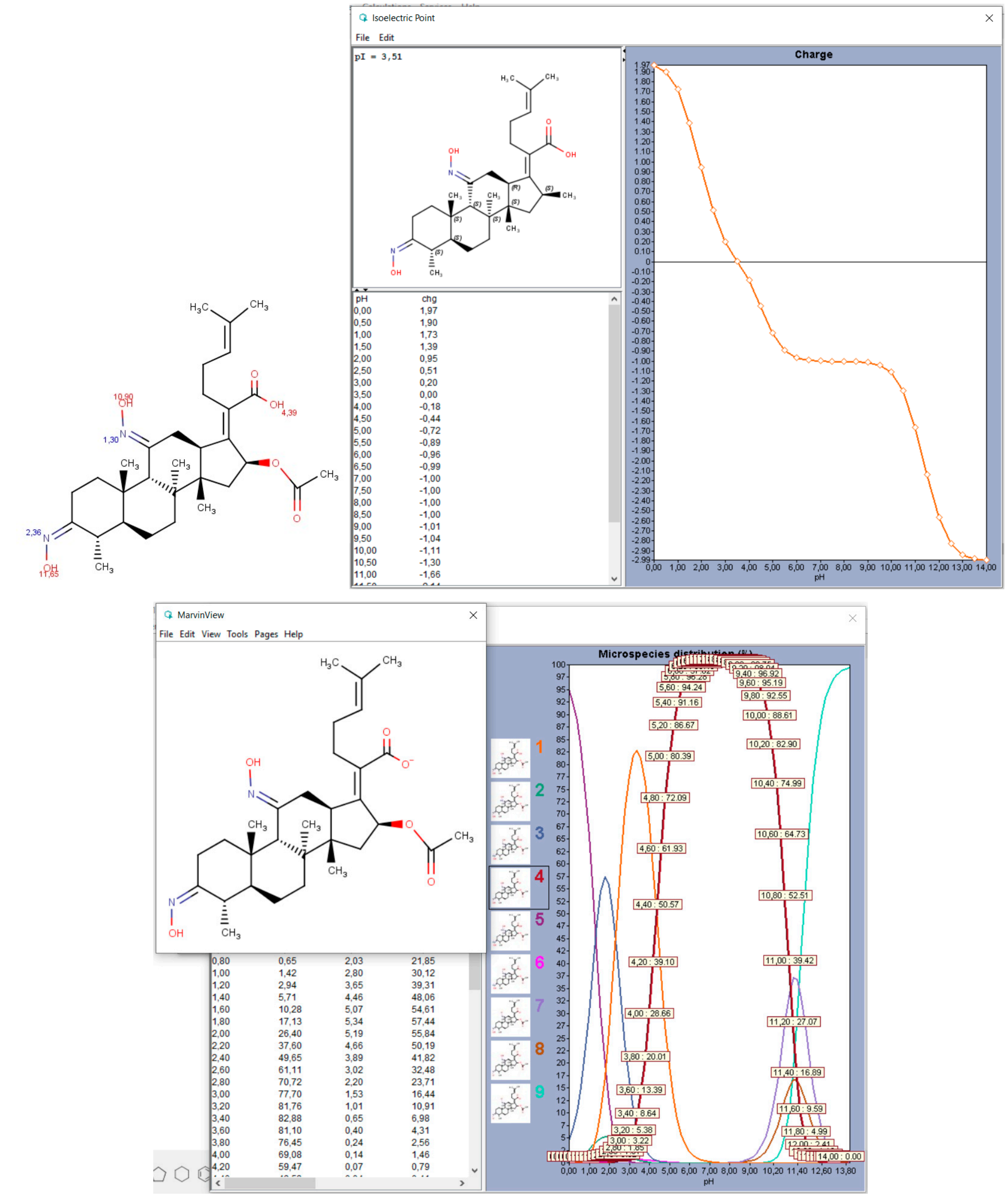

Figure S5. Theoretical values of pKa and pI for compound **1**.

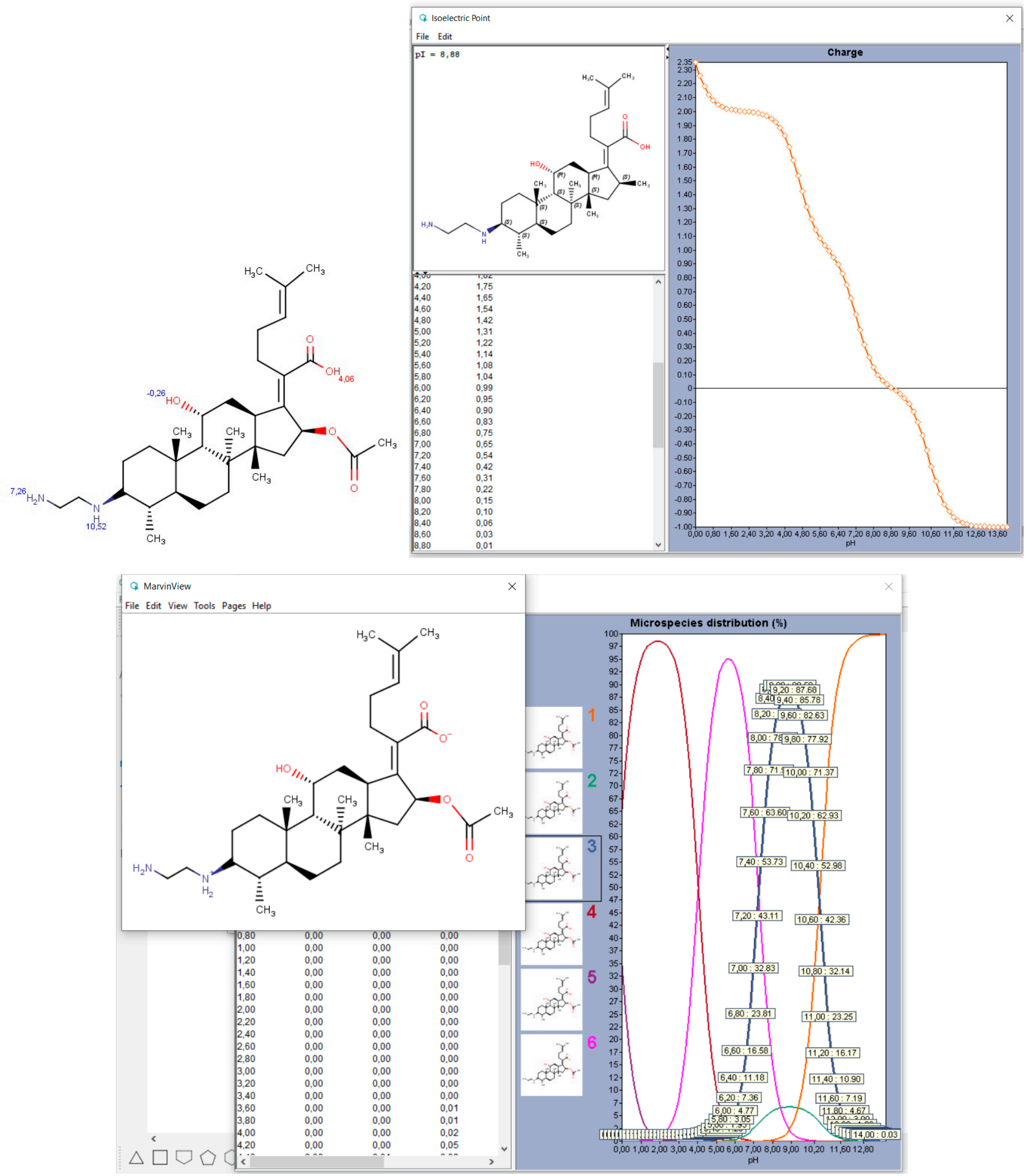

Isoelectric Point
File Edit
pI = 8,88
Charge
pH
MarvinView
File Edit View Tools Pages Help
Microspecies distribution (%)
pH

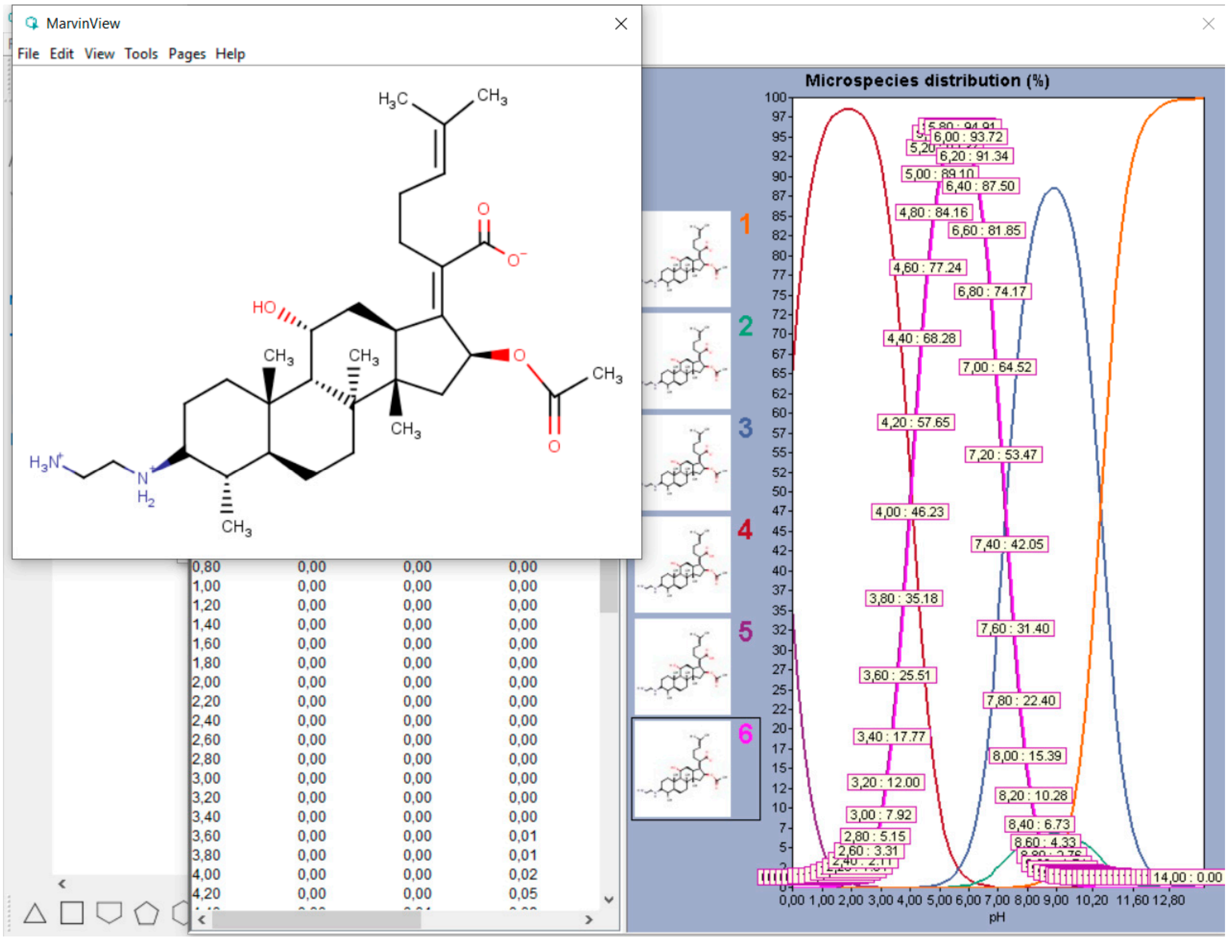

Figure S6. Theoretical values of pKa and pI for compound **2**.
